# A Digital Behavioral Activation Intervention (JuNEX) for Pregnant Women With Subclinical Depression Symptoms: Explorative Co-Design Study

**DOI:** 10.2196/50098

**Published:** 2024-05-16

**Authors:** Elisa Mancinelli, Silvia Gabrielli, Silvia Salcuni

**Affiliations:** 1 Department of Developmental and Socialization Psychology University of Padova Padova Italy; 2 Fondazione Bruno Kessler Trento Italy

**Keywords:** digital intervention, behavioral activation, feasibility, pregnancy, subclinical depression symptoms

## Abstract

**Background:**

Digital interventions are gaining increasing interest due to their structured nature, ready availability, and self-administered capabilities. Perinatal women have expressed a desire for such interventions. In this regard, behavioral activation interventions may be particularly suitable for digital administration.

**Objective:**

This study aims to exploratorily investigate and compare the feasibility of the internet-based self-help guided versus unguided version of the Brief Behavioral Activation Treatment for Depression-Revised, an empirically supported in-person behavioral activation protocol, targeting pregnant women with subclinical depression symptoms. A user-centered design is used, whereby data are collected with the intent of evaluating how to adjust the intervention in line with pregnant women’s needs. Usability and user engagement were evaluated.

**Methods:**

A total of 11 Italian pregnant women with subclinical depressive symptoms based on the Patient Health Questionnaire-9 (scoring<15) participated in this study; of them, 6 (55%) women were randomly assigned to the guided group (age: mean 32.17, SD 4.36 years) and 5 (45%) to the unguided group (age: mean 31, SD 4.95 years). The Moodle platform was used to deliver the interventions in an e-learning format. It consisted of 6 core modules and 3 optional modules; the latter aimed at revising the content of the former. In the guided group, each woman had weekly chats with their assigned human guide to support them in the homework revisions. The intervention content included text, pictures, and videos. Semistructured interviews were conducted, and descriptive statistics were analyzed.

**Results:**

Collectively, the data suggest that the guided intervention was better accepted than the unguided one. However, the high rates of dropout (at T6: guided group: 3/6, 50%; unguided: 4/5, 80%) suggest that a digital replica of Behavioral Activation Treatment for Depression-Revised may not be feasible in an e-learning format. The reduced usability of the platform used was reported, and homework was perceived as too time-consuming and effort-intensive. Moreover, the 6 core modules were deemed sufficient for the intervention’s goals, suggesting that the 3 optional modules could be eliminated. Nevertheless, participants from both groups expressed satisfaction with the content and found it relevant to their pregnancy experiences.

**Conclusions:**

Overall, the findings have emphasized both the intervention’s merits and shortcomings. Results highlight the unsuitability of replicating an in-person protocol digitally as well as of the use of nonprofessional tools for the implementation of self-help interventions, ultimately making the intervention not feasible. Pregnant women have nonetheless expressed a desire to receive psychological support and commented on the possibilities of digital psychosocial supports, particularly those that are app-based. The information collected and the issues identified here are important to guide the development and co-design of a more refined platform for the intervention deployment and to tailor the intervention’s content to pregnant women’s needs.

## Introduction

### Background

Peripartum depression refers to an episode of depression that meets the criteria for persistent or major depressive disorder, with onset occurring during the peripartum period [1]; this definition highlights the direct link between the development of the depressive condition and the bodily changes and overall characteristics inherent to the perinatal period [2]. A distinction should also be made with regard to antenatal and postnatal depression, since antenatal depression is recognized as one of the main predictors of postnatal depression, with the latter then aggravating the repercussions on the mother as well as on the child and the whole family [3]. In Europe, antenatal depression counts a mean prevalence of 17.9% [4], while postnatal depression ranges from an average of 12.91% to 16.62% [5]. In Italy, specifically, the literature highlights a prevalence ranging from 6% to 22% for antenatal depression [6-9] and from around 13% to 23% for postnatal depression [5,7,9,10]. Such percentages emphasize the necessity for early detection and the implementation of prevention programs to alleviate depression symptoms already during pregnancy. Notably, women have expressed a desire for perinatal support programs and have reported benefiting from them, both in terms of symptom reduction and increased sense of agency [11]. However, barriers to help seeking, in the context of perinatal care, have been widely recognized (*knowledge barriers*, eg, difficulty in recognizing health needs and distinguishing emotional difficulties as well as not knowing the services available; *practical barriers*, eg, time and economic constraints; and *attitudinal barriers*, eg, stigma, guilt) [11-13], contributing to the still limited availability of the services to support perinatal women’s mental health [14].

Within this context, digital solutions might be particularly valuable. A recent review that specifically focused on the application of eHealth in perinatal care [15] highlighted the potential of these solutions as alternatives or supplements to standard mental health practices, both for screening and intervention. A subsequent review [16] also emphasized the beneficial role of digital solutions in addressing perinatal depression by enhancing accessibility to psychological interventions, thus promoting scalability, which could ultimately allow to work around the abovementioned barriers to help seeking. Digital interventions could thus be valuable solutions to fill in the gap between what is asked and desired by women and the logistic and economic limits on both clinical professionals and health institutions part. Notwithstanding, despite the increasing focus on peripartum depression, there is currently a scarcity of digital psychological interventions aimed at alleviating depression symptoms, particularly those grounded in empirically validated intervention protocols [16-18]. Furthermore, the prevailing focus appears to lean more toward treating rather than preventing perinatal depression. This is evident in the dearth of studies investigating digital interventions during pregnancy and exemplified by the lack of studies investigating digital interventions deployed during pregnancy and to women with subclinical depression symptoms [17,18]. In light of this, there is a need to develop theoretically grounded digital interventions tailored to pregnant women with subclinical symptoms needs and characteristics, ultimately preventing the development or worsening of clinically relevant depression symptoms during the postpartum.

Evidence-based interventions that are brief and structured such as behavioral activation (BA) interventions might be especially helpful to this end, as providing pregnant women with concrete strategies will be useful to support their adjustment. BA is an empirically supported behavioral intervention created to lessen depression symptoms [19-22]; it is based on the idea that a greater awareness of the mutual influence between behavior and emotion can ultimately encourage behavioral change by increasing participation in joyful and adaptive activities while reducing participation in maladaptive behaviors that maintain or exacerbate the depressive symptoms [19,23]. However, a recent scoping review [18] highlighted a gap in the literature: there are few digital BA interventions available during the perinatal period, and none have been specifically deployed during pregnancy. Furthermore, their usability has only been marginally evaluated.

Usability refers to the quality of the interaction occurring between the user (eg, pregnant women) and the tool used (eg, website, smartphone app, etc) [24]; a subcomponent of usability that is more specific is the *user engagement*, which includes the user’s cognitive, behavioral, and affective reaction to the tool [25]. These factors are instrumental in supporting user compliance and adherence, and they should be carefully considered and addressed in the development of feasible and acceptable digital interventions [26]. The limited evaluation of these factors in the context of digital mental health solutions may be attributed to the novelty of the field, which has yet to establish a comprehensive understanding of design methods for such tools [27]. When designing these digital solutions, four components should be kept in mind: (1) the design issue and solution, (2) the context in which the design occurs, (3) the dynamics and organization of the design activity, and (4) the actors contributing to the design [28-30]. However, a recent review [27] investigating the design methods and approaches used for the design and development of digital tools for mental health stressed that *human-centered design methods* (ie, the design of digital tools not considering the engineering design and including user-centered approaches, co-design, participatory design, etc) are not yet fully integrated within the field and that reported design approaches are still mainly external, thus excluding the perspective of those for whom the tool is created.

### This Study

This study aimed to investigate the feasibility of the Brief Behavioral Activation Treatment for Depression-Revised (BATD-R) [31] protocol that was structured as an internet-based self-help intervention and deployed to pregnant women with subclinical depression symptoms. Compared with other BA protocols, the BATD-R protocol specifically targets subclinical depression symptoms, is flexible (in terms of both its structuring and the population it is administered to), and can be self-administered or deployed by both specialists or nonspecialists [31].

Given that no previous study has used the BATD-R protocol for this purpose, the intervention developed and evaluated in this study serves as digital “replica” of the in-person BATD-R protocol. By adopting a *user-centered* approach, this study not only aimed to assess its initial feasibility but also sought to gather valuable feedback directly from pregnant women. This feedback will guide the adjustment of the intervention’s content and thus its structure, without making assumptions beforehand about the changes required. Indeed, a user-centered design “is an approach to product development that grounds the process in information about the people who will ultimately use the product” [32]; as such, to create a well-accepted, engaging, and effective digital intervention in perinatal care, subsuming the intervention content and the mean through which it is deployed, pregnant women should be consulted in each stage of the intervention’s creation and refinement, thereby ultimately allowing the co-design of the final intervention. In this regard, this study relied on the Obesity-Related Behavioral Intervention Trials model [33], which provides an iterative progressive framework guiding the development, testing, and refinement of the behavioral intervention. More specifically, it uses a user-centered design that relies on a data-driven approach to iteratively test and revise the intervention, up until it is deemed appropriate to move to further phases of development and testing, thereby going from the intervention design, its preliminary testing to investigating its efficacy and effectiveness [33].

As previously reported, no digital BA intervention targeting subclinical or clinical depression symptoms among pregnant women has been developed [18]. Nonetheless, it is worth noting that among the existing digital BA interventions, many are guided interventions [18]. The guides, most often mental health specialists or trained professionals, provide additional support to women throughout the intervention, by addressing concerns or supporting them on intervention-related tasks. Mindful of this, a further aim of this study was also to explore and evaluate the role and potential benefits of including a guide as additional support in the self-help intervention. As such, the study also aimed to compare the feasibility of the guided versus unguided version of the intervention.

## Methods

### Recruitment

**Figure 1:**
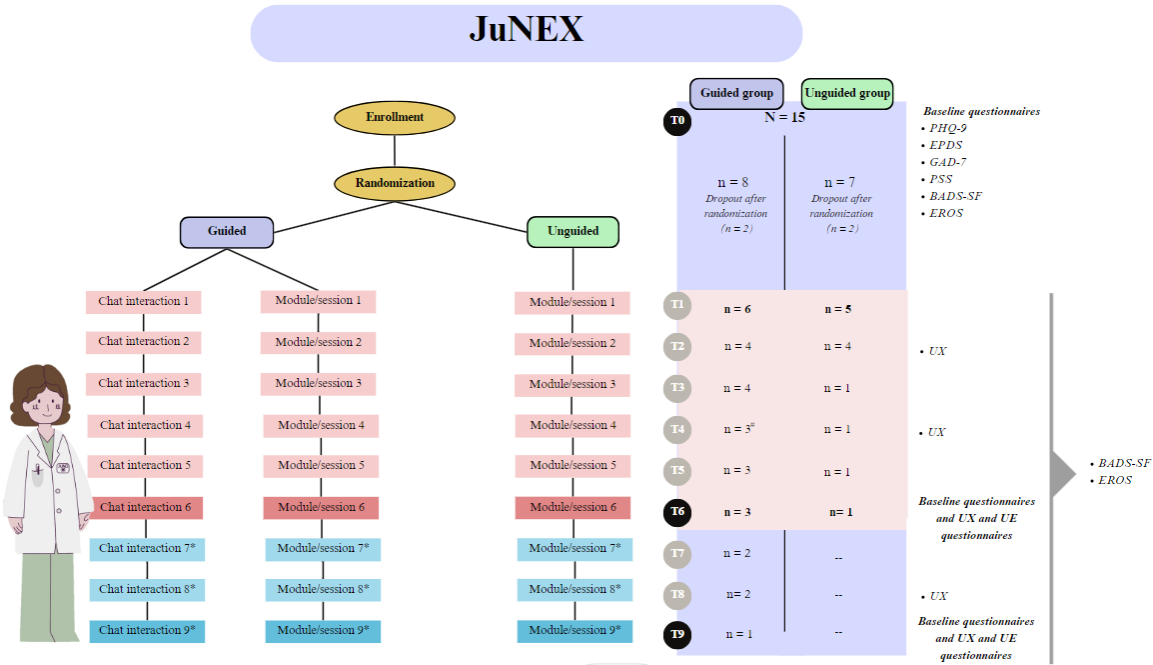
Study structure and study adherence flowchart. *Indicates optional modules and # indicates that 1 participant had stopped interacting with the guide but had continued viewing the Moodle content. BADS-SF: Behavioral Activation for Depression Scale–Short Form; EPDS: Edinburgh Postnatal Depression Scale; EROS: Environmental Reward Observation Scale; GAD-7: Generalized Anxiety Disorder-7; PHQ-9: Patient Health Questionnaire-9; PSS: Perceived Stress Scale; T: time point; UE: user engagement; UX: user experience.

Recruitment was done through snowball sampling, using social media platforms (eg, Facebook). A Google Form survey was developed containing the informed consent and the questions and questionnaires needed to evaluate the women’s eligibility for participating in the study. Specifically, eligible women complied with the following inclusion criteria: they (1) had physiological pregnancy, (2) were aged ≥18 years, (3) were between the 12th and 30th week of gestation, and (4) had subclinical depression symptoms (Patient Health Questionnaire-9 [PHQ-9] score<15) [34]. By contrast, women were excluded when (1) presenting a history of past or current mental disorders; (2) exhibiting clinically significant psychological symptoms (ie, depression symptoms: PHQ-9≥15) and suicidal ideation (PHQ-9 item 9); (3) having an obstetrically at-risk pregnancy; (4) presenting medical conditions, pregnancy-related and otherwise; (5) experiencing an artificially induced pregnancy. A total of 15 women had filled in the web-based questionnaire; all were deemed eligible, and thus none reported any of the exclusion criteria. Following randomization in either the guided or unguided group, 4 (27%) women dropped out (Figure 1) before starting the intervention. As such, the final sample is composed of 11 (73%) women.

### Procedure and Study Structure

This study is structured in 4 main phases (Figure 1). Phase 1 corresponds to time point 0 (T0), which encompasses the recruitment, enrolment (including the assessment of inclusion and exclusion criteria), and randomization processes. At this time, anamnestic information (reported in the *Recruitment* section) was collected, and standardized questionnaires were administered measuring depression and anxiety symptoms, perceived stress, current activity level, and perceived environmental reward. Phases 2 and 3 of the study correspond to T1-T6 and T7-T9, respectively. During these phases, the baseline questionnaires, along with one questionnaire assessing user engagement (UE) and another assessing user experience (UX; explained in the *Measurement Tools* section) were administered. In addition, the UX questionnaire was also administered during T2, T4, and T8. Questionnaires assessing the level of activity and related reward (explained in the *Measurement Tools* section) were administered each week, from T0 to T9. The final phase 4 involved conducting semistructured interviews that were created ad hoc, and participants who had participated up to at least T6 were interviewed. The semistructured interviews were conducted to qualitatively evaluate the women’s experience with the intervention and gather further feedback necessary for refining the intervention. All questionnaires administered between T1 and T9 were created using Google Forms. Links to access these questionnaires were made available within the intervention platform and were accessible to both groups in the same manner.

The participants were informed that they could leave the study at any moment without having to provide an explanation and without incurring in any penalty. Each was assigned an alphanumeric code to ensure confidentiality.

### Randomization

Alphanumeric codes were generated and allocated to each participating woman to guarantee confidentiality throughout the study. The process of creating these codes was carried out using Microsoft Excel and further randomized through a Google software [35], ensuring the unbiased assignment of participants to either the guided or unguided group before the commencement of recruitment. The results of random code assignment were kept aside, and women were only provided with their designated code once their eligibility for the study had been established. Only when eligibility was confirmed, participants were given specific information on the intervention they had to follow. The group assignment was single blinded.

### The Intervention’s Content

This internet-based self-help intervention originates from the BATD-R [31] protocol. Originally, this protocol consisted of 10 sessions, which included 5 main sessions and 5 additional sessions aimed at reviewing and maintaining the benefits achieved; nevertheless, it is possible to reduce the number of sessions to 5 or even expand beyond 12 sessions. However, past studies advise against exceeding 10 weeks of intervention, reporting that BATD-R interventions of up to 6 or 8 weeks allow for a significant reduction in depression symptoms [36,37]. BATD-R can be self-administered or administered by both trained and untrained staff, further emphasizing its adaptability and flexibility. The protocol begins with a psychoeducation phase focused on understanding the characteristics of depression symptoms. Furthermore, it includes homework assignments to be completed between sessions, which form the core content of the 5 main sessions. These assignments involve filling out 5 forms (refer to the original protocol by Lejuez et al [31]). The first form, called the “daily monitoring form,” should be completed throughout the intervention and requires participants to continuously evaluate their daily activities, in terms of behavioral patterns, the pleasantness of activities, and the importance of each daily action. Subsequently, the person is required to identify through a second form, called “life areas, values, and activity inventory,” what their values are within important areas of life (ie, important relationships; pleasurable activities and hobbies; work and study; mind, body, and spirituality; and daily responsibilities). This would then allow the person to identify (form 3, called “activity selection and ranking”) and plan daily activities (form 4, called “action plan”) that help them live in accordance with their values in the mentioned life areas. In this regard, it is worth noting that although the BATD-R does not include a complete functional analysis due to the brevity of the treatment approach [38], Lejuez et al [31] stressed that several components of treatment fit into a functional analytical framework. This is most noticeable when choosing activities that are closely related to values, given the dual goals of identifying the factors that maintain or reinforce depressive behavior (both positive and negative reinforcement) and the positive reinforcers that could support or strengthen healthy behavioral patterns. Along with the critical assessment of dysfunctional behavioral patterns, the BATD-R protocol includes a final form called “contracts,” which focuses on strategies to request social support. To create these “contracts,” the person is required to identify (1) an activity to perform, (2) up to 3 support persons who may be able to assist or support them, and (3) how and when each person can specifically provide said support. This activity allows the patient to identify their needs and provides a specific plan on how to seek the help they need by making concrete requests for obtaining assistance and social support to reinforce adaptive behaviors.

In this study, the intervention closely follows the structure of the original protocol but is adapted to fit a digital format. The intervention was divided into 6 weekly sessions or modules, which include the core content of the intervention. In addition, 3 optional “bust” sessions were included to reinforce and consolidate the information from previous weeks. The entire intervention spanned 9 weeks, with participants completing weekly homework assignments between sessions. While the intervention content and homework remained unchanged, it was adapted for digital delivery using text, video, and images. Information related to depression symptoms was contextualized for the pregnancy period to cater to the specific needs of the target population.

### The Platforms

#### Overview

In this first evaluation of the intervention, the Moodle e-learning platform (Moodle 3.11; 2021) was used as the delivery method. This platform was accessible via both the web and the Moodle app on smartphones. Both the web and app versions of Moodle included a chat feature, which was exclusively used by the guided group to interact with their assigned guides. Specifically, in the guided group, guide-woman dyads were created, and they interacted once a week for the homework revision. For the unguided group, homework revision was facilitated through a written self-guide available within the Moodle platform. The forms representing the homework were structured on Google Docs, with links to access them made available within the Moodle platform so that women could directly access them both on the web and the smartphone app.

Both intervention groups were presented with the intervention as an e-learning Moodle course; the sole difference in the intervention’s content between the groups lay in the reference to the guides. The intervention was structured into modules (6 core modules and 3 optional modules), and each module could be consulted only after completing the previous one. The content of each module was delivered using illustrative videos and images, complemented by brief text information. After viewing each section within a module, participants were presented with a brief quiz comprising 3 true-or-false questions related to the content they had just reviewed. These quizzes were incorporated with the intention of fostering UE. They encouraged participants to actively engage with the material rather than to passively view it. In addition, the quizzes served as a means of assessing participants’ comprehension of the content. On the basis of the accuracy of their responses, participants received reinforcing or motivating feedback after completing each quiz.

#### The Guides

In the guided group, specific guide-woman dyads were randomly created. All the guides were recognized psychologists who had been trained to become psychotherapists. They underwent comprehensive training, which included the provision of detailed written information and a 2-hour in-person meeting. This training covered the intervention’s content and structure and their role as guides. The training aimed to ensure that all the guides had a consistent understanding of the intervention and its content, thus maintaining uniformity across the interactions between the guides and participants. The guides adhered to a partially defined conversational protocol, which consisted of fixed messages and information to be delivered, as well as “free” parts where they had the freedom to phrase sentences as they saw fit. This flexibility in the “free” parts allowed guides to respond adaptively to women’s answers and feedback, particularly during the homework revision part of the intervention. Furthermore, it enabled guides to support participant compliance and adherence based on their perceived motivation levels. Supervision for the guides was provided by the first author (EM) of the study, who oversaw the technical aspects of the intervention. In addition, an expert psychotherapist, the third author (SS), provided supervision for clinical matters.

To ensure a consistent participant experience, all the guides were assigned the same name, “Joy.” This uniformity aimed to minimize any potential biases that could arise from variations in the perception of the different guides. Moreover, guides were not provided with any information about the women they were assigned to, ensuring privacy and confidentiality for the participants.

### Measurement Tools

#### PHQ-9 Tool

The PHQ-9 [34] is a unidimensional self-report tool that is widely used in the Italian context [39]. It assesses the severity of depression symptoms during the previous 2 weeks, based on the diagnostic criteria of the *Diagnostic and Statistical Manual of Mental Disorders* (*Fourth Edition*; *DSM-IV*) [40]. It consists of 9 items measured on a 4-point Likert scale (0=“not at all”; 3=“almost every day”). Item 9 assesses suicidal ideation. A score of ≤9 indicates mild or no symptoms of depression, and a score between 10 and 14 indicates moderate symptoms, while a score of ≥15 indicates severe symptoms of depression. The instrument shows excellent internal consistency at α=.92 [41].

#### Edinburgh Postnatal Depression Scale

Edinburgh Postnatal Depression Scale [42] is a unidimensional self-report tool, validated in Italy [43], which assesses the severity of depression symptoms during the previous week. Albeit developed to assess depression symptoms during the postpartum period, it is often used throughout the perinatal period. It consists of 10 items measured on a 4-point Likert scale (0=“no, not at all”; 3=“yes, always”). The instrument shows good internal consistency at α=.79 [43].

#### Generalized Anxiety Disorder-7

Generalized Anxiety Disorder-7 [44] is a unidimensional self-report tool that assesses the severity of anxiety symptoms during the previous 2 weeks. It consists of 7 items measured on a 4-point Likert scale (0=“never”; 3=“almost every day”) and shows good psychometric indexes in the Italian context as well [41]. The instrument shows excellent internal consistency at α=.92 [41].

#### Perceived Stress Scale

Perceived Stress Scale [45] is a unidimensional self-report tool, validated also in Italy [46], which assesses the severity of stress symptoms in the previous month. It consists of 10 items measured on a 4-point Likert scale (0=“never”; 3=“quite often”). The instrument shows good internal consistency at α=.74 [46].

#### Behavioral Activation for Depression Scale—Short Form

Behavioral Activation for Depression Scale–Short Form [47] is a self-report tool designed to measure changes in avoidance and activation during BA interventions for depression during the previous week. It consists of 9 items measured on a 7-point Likert scale (0=“not at all”; 6=“completely”). The scale provides 2 scores, the first score referring to the level of BA (5 items) and the second one to the level of behavioral avoidance (5 items). Manos et al [47], the authors of the tool, advise considering the total score instead of the subscales. This questionnaire has not been translated into Italian and was therefore translated through the back translation procedure. Example items are “I am content with the amount and types of things I did” (item 2) and “Most of what I did was to escape from or avoid something unpleasant” (item 5). The instrument shows good internal consistency (total scale α=.82) [47].

#### Environmental Reward Observation Scale

Environmental Reward Observation Scale [48] is a unidimensional self-report tool designed to measure the level of environmental reward perceived in recent months. It consists of 10 items rated on a 4-point Likert scale (0=“strongly disagree”; 4=“strongly agree”). This questionnaire has not been translated into Italian and was therefore translated through the back translation procedure. Example items are “It is easy for me to find enjoyment in my life” (item 4) and “I wish that I could find more hobbies that would bring me a sense of pleasure” (item 7). The instrument shows good internal consistency (α=.87) [48].

#### UX Measure

Mobile Application Rating Scale (MARS) [49] is a self-report tool consisting of 23 items scored on a 5-point Likert scale (1=“poor”; 5=“excellent”), which assesses the quality of the app and its features (ie, the Moodle app) on 4 dimensions of objective quality: engagement (5 items), functionality (4 items), aesthetics (3 items), and information (7 items); a final scale assesses the subjective quality (4 items). The average of the scores of the 4 dimensions of objective quality provides the total scale score. The questionnaire also contains an “application-specific” section (6 items) to assess the potential impact of a particular app on domains such as users’ knowledge and intentions. The total and subscale scores of the MARS have high internal consistency coefficients (α=.90 and α=.80-.89, respectively). The scale has been validated in the Italian context [49]. For this study, only the subscales related to “information,” “subjective app quality,” and “app-specific” sections were considered, totaling to 17 items.

Together with the MARS items, only at T6 and T9, women were also asked to prove their overall subjective opinion on the platform (“Please write below your personal opinion with respect to your experience [pros and cons] while using the platform”).

#### UE Measure

UE Scale–Short form [25] is a short self-report tool designed to assess UE with a digital solution. It consists of 12 items based on a 5-point Likert scale (1=“strongly disagree”; 5=“strongly agree”). The questionnaire consists of four factors: (1) focused attention, which indicates the feeling of being immersed in the interaction; (2) perceived usability, which is the negative effect experienced due to the interaction and the effort expended; (3) aesthetic attractiveness, which represents the graphical and visual appeal concerning a digital solution; and (4) the reinforcement (reward) factor, which regards the perceived involvement and enjoyment with the digital solution. This questionnaire was not translated into Italian and was therefore translated through the back translation procedure. The 4 scales have good internal reliability, as follows: focused attention, ω=0.75; perceived usability, ω=0.70; aesthetic attractiveness, ω=0.88; and reinforcement, ω=0.79 [25].

#### Semistructured Interview

The semistructured interviews were conducted by the first author (EM) and featured 15 main questions developed specifically for the study, 3 (20%) of which were asked only to the guided group. This interview was conducted approximately 10 days after each woman had finished the intervention. It lasted between 15 and 20 minutes, and following the woman’s consent, it was audio recorded to allow for its transcription and evaluation. For both the guided and unguided groups, the interviews investigated women’s personal experience (7 questions) with the intervention and the experience (5 questions) specifically related to the use of the platform. In the guided group, women’s experience with their guide and the overall chat interactions were investigated.

### Data Analysis

Statistical analyses were performed using RStudio (R Foundation for Statistical Computing) [50]. Descriptive data for both categorical (n, %) and continuous (mean and SD) variables were analyzed separately for the guided and unguided groups, considering the different time points. Given the preliminary nature of the study and the small sample size, no further analyses were performed. The interviews were individually and qualitatively analyzed through thematic analysis, following the predefined semistructured interview’s 3 broader themes. Thematic analysis was conducted following a modified version of the guidelines proposed by Braun and Clarke [51], which has already been used in other co-design studies [52].

### Ethical Considerations

The study was conducted in compliance with the ethical guidelines of the Declaration of Helsinki [53] and the European Union law for data protection (EU General Data Protection Regulation 679/2016). The study was approved by the Ethical Committee of the Psychology Department of the University of Padova (number 4820/2022).

## Results

### Descriptive Information and Adherence

A total of 11 women participated in the study; 6 (55%) were part of the guided group and 5 (45%) were part of the unguided group. Descriptive information is reported in Table 1 separately for the 2 groups.

**Table 1:** Descriptive information (n=11).

		Guided group (n=6)	Unguided group (n=5)
Age (years), mean (SD)		32.17 (4.36)	31 (4.95)
Gestation week, mean (SD)		21.17 (5.95)	19.83 (5.75)
**Living area, n (%)**			
	North Italy	4 (67)	4 (80)
	Central Italy	2 (33)	1 (20)
	South Italy	0 (0)	0 (0)
**Education, n (%)**			
	<High-school diploma	1 (17)	0 (0)
	High-school diploma	1 (17)	2 (40)
	Bachelor degree	1 (17)	0 (0)
	Master degree	2 (33)	2 (40)
	Specialization (eg, PhD)	0 (0)	1 (20)
**Marital status, n (%)**			
	Single	0 (0)	0 (0)
	Cohabitant	3 (50)	2 (40)
	Married	3 (50)	3 (60)
**Past abortion, n (%)**			
	Yes	2 (33)	1 (20)
	No	4 (67)	4 (80)
**Women’s occupation, n (%)**			
	Unemployed	1 (17)	0 (0)
	Student	1 (17)	0 (0)
	Student and freelance worker	1 (17)	0 (0)
	Employee	3 (50)	3 (60)
	Student and employee	0 (0)	1 (20)
	Researcher	0 (0)	1 (20)

The descriptive statistics pertaining to psychosocial variables assessed at T0, T6, and T9 for both groups are presented in Table 2.

**Table 2:** Descriptive statistics at time point 0 (T0), T6, and T9 (n=11).

	Scores of the guided group (n=6)			Scores of the unguided group (n=5)	
	T0 (n=6), mean (SD)	T6 (n=3), mean (SD)	T9 (n=1)	T0 (n=5), mean (SD)	T6 (n=1)
PHQ-9^a^	6.67 (2.66)	3.33 (2.08)	3	3.4 (2.79)	1
EPDS^b^	15 (2.37)	11 (3.61)	6	10.6 (3.21)	9
GAD-7^c^	7 (1.41)	5 (2)	3	4.0 (1)	3
PSS^d^	20.50 (2.07)	18.67 (2.08)	17	18.0 (2.3)	17
BADS-SF^e^	23.17 (8.18)	16.67 (1.15)	18	19 (2.92)	27
EROS^f^	28.17 (4.17)	30.67 (2.52)	34	32 (3.39)	36

^a^PHQ-9: Patient Health Questionnaire-9.

^b^EPDS: Edinburgh Postnatal Depression Scale.

^c^GAD-7: Generalized Anxiety Disorder–7.

^d^PSS: Perceived Stress Scale.

^e^BADS-SF: Behavioral Activation for Depression Scale–Short Form.

^f^EROS: Environmental Reward Observation Scale.

Regarding dropout rates, it was higher in the unguided group (n=5), with most participants (n=4, 80%) dropping out after completing the second module. In contrast, the dropout pattern in the guided group (n=6) was more gradual. Overall, 1 (9%) participant had dropped out because of health reasons and 3 (27%) because of the amount of time and effort (particularly related to the homework) required by the intervention, while 3 (27%) did not provide a reason for dropping out. Accordingly, 3 (50%) participants from the guided group reached T6, thereby completing the 6 core modules in Moodle, while only 1 (20%) in the unguided group reached T6. However, among the 3 participants from the guided group who reached T6, 1 (33%) ceased interactions with her guide after T3 but continued viewing the material on Moodle up to T6; the other 2 (67%) continued until T9. One of them stopped viewing the Moodle content at T8 but continued with the chat interactions with the guide.

Regarding homework completion, it is not possible to quantify adherence specifically, as, for instance, among the participants that had completed at least until T6 some (2/4, 50%) decided to handwrite the homework, instead of using Google Docs because of the low usability of the latter (ie, too many steps to go from Moodle to Google docs, which were also not well perceived and difficult to use within the smartphone). Moreover, it was considered cumbersome to write within the Google Docs.

### UX and UE Measures

**Figure 2:**
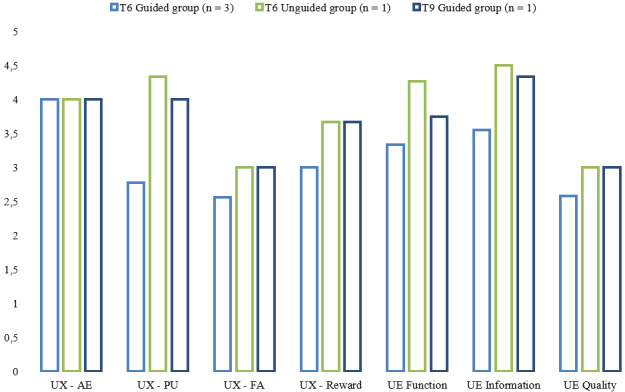
User experience (UX) and user engagement (UE) at time point 6 (T6) and T9. All scales’ response range was from 1 to 5. AE: aesthetic appearance; FA: focused attention; PU: perceived usability; quality: subjective quality.

Descriptive statistics for UX and UE assessed at T6 and T9 are illustrated in Figure 2. At T6, when the participants were asked whether they had used primarily the app or web version of Moodle, 2 (67%) participants of the guided group and the only 1 participant of the unguided group reported using the app version, while 1 (33%) of the participants of the guided group used primarily the web version because of difficulties with using the app.

Participants’ subjective opinion on the platform, assessed through one open question, is reported in Table 3.

**Table 3:** Answers to the open question “Please, write below your personal opinion with respect to your experience (pros and cons) using the platforms.”

Time points and group			Participants’ feedback
**Time 6**			
	Unguided group, n=1	“I am in my second pregnancy so I have been using the platform with a one-and-a-half-year-old [taking care of them] and I must say that being able to subdivide the time has been helpful even on an organizational level. If one fills out the daily forms from day to day it is not challenging, I must say that filling them out [the daily forms] from your cell phone though is quite inconvenient because the app [Google Docs] that opens the forms and allows you to fill them out does not always work well, so I then preferred to print them out and fill them out by hand.”	
	Guided group, n=3	“I think it is still very cumbersome as a platform [Moodle], not easy to use and the interaction with the Guide [was] too dry. Some things need to be revised.” “Using the platform on a practical level was quite intuitive and easy. The project itself involves a lot of effort and concentration, but it helps to feel a greater physical and psychological well-being.” “It was intuitive and fast.”	
**Time 9**			
	Guided group, n=1	“My experience in using the platform has been positive.”	

### Semistructured Interviews Results

A total of 3 participants agreed to participate in the semistructured interview; 2 (67%) were from the guided group and had continued the intervention till T9, while 1 (33%) was from the unguided group and had participated till T6.

During the semistructured interview, all 3 (100%) participants reported that they had come to know of the study through a friend. As motivation for participating, all 3 reported “*curiosity*” as the main reason because they did not have specific expectations before starting.

Overall, participants expressed that the intervention helped them find a moment for themselves and provided them with a method or strategy to change their perspective on how they viewed and performed their daily activities. Furthermore, they noted the positive effects of engaging in more rewarding activities. All 3 (100%) participants emphasized that the intervention supported their overall well-being rather than reduced negative feelings per se. However, they also mentioned that the effort required by the intervention, particularly in terms of time and dedication, was substantial, especially regarding the homework assignments. While they appreciated the meaningful content of the homework, they found it burdensome to complete. It is important to note that part of the difficulty with homework completion was related to the reduced fluidity and usability of Google Docs on smartphones. A more thorough explanation of the findings that emerged from the semistructured interviews with verbatim examples of participants’ answers has been discussed as follows, and the specific themes and subthemes that emerged are reported in Table 4.

**Table 4:** Semistructured interview (n=3): themes, subthemes, and examples.

Theme and subtheme			Example quotes
**Personal experience with the intervention**			
	Self-observation and activities evaluation	“[...]you realize things that by not doing it [the intervention] you wouldn’t have realized you could have done, how you could have also handled the pregnancy period better” [Unguided group participant] “It definitely helped me understand what are [...] let’s say, [which is] the focus, where to aim to get better. I discovered some things that I had set aside[...] it helped me to not be so focused on negative things, which is kind of my problem, but more to identify something positive to do day-by-day, to be able to accomplish little goals that [might not be important for others] but for me at that moment they were important” [Guided group participant] “[...]most definitely [I appreciated] focusing my attention on some positive activities that I had somewhat set aside and forgotten[...] when I focused on those saddest moments and I [then] realized that they were not most of my days as one thinks when one is in the sad mood. Instead, I saw that most of my days were good moments” [Guided group participant]	
	Effort	“As the weeks went on, maybe even as the pregnancy progressed[...] I found it more ‘burdensome.’ The fact of filling out the daily forms [daily monitoring form] every day[...]. as time went on, it was challenging, in the sense that one has to stop and really take [their] time and be consistent, when as the pregnancy progresses maybe other thoughts take over and you can’t quite be that consistent all the time[...]” [Guided group participant] “[...]the part, let’s say the most obnoxious, difficult, whatever we want to call it, is definitely the material to fill out during the last weeks. I have to tell you the truth, I didn’t even finish them because I didn’t have time[...]” [Guided group participant] “In my experience[...]. I felt the fatigue more, maybe, here. Let’s say that at the end of the six (sixth module), for myself, I felt that the intervention was -in quotes- “finished.” [Guided group participant] “[In reference to the intervention length] it probably depends on what stage of the pregnancy one is[...] I started it toward the end of my pregnancy, it would probably be better to start it before[...]”[Unguided group participant]	
	Learning the “method” and its application in the future	“[...]It might be a good method at other times in life when one may experience difficulties” [Guided group participant] “[...]mentally, I got into this mode of planning something nice to do, to be able to have that weekly commitment that I like, to ask somebody to do it with me. Maybe small things, but I’m sure it helped me for the future as well.” [Guided group participant]	
**User experience and user engagement**			
	Managing issues and learnability	“[...]when I had to write [for the homework], there was the transition from the daily monitoring [form] rather than very often I had trouble writing things down[...] I mean, I had to print them out [the forms] basically, if not I couldn’t record [write] them [down].” [Unguided group participant] “[A]t first maybe you kind of have to learn the mode of, yeah, that you get the materials out[...] It wasn’t easy to tell when it saved what you had done, because it was a little dubious, sometimes[...] it was easy to use once you had gotten the hang of it, going into the week, looking at the material...” [Guided group participant]	
	Multimedia material	“[...]the videos etc. were very effective in passing the message, both in terms of explanations and content, and the images, they were[...] they caught the attention[...]” [Guided group participant] “[T]he videos[...] they are very clear, well done, they are cute” [Unguided group participant]	
	App interventions in the future	“[...]with the current use of the phone and the computer in general, in my opinion an app is useful” [Unguided group participant] “[...]the app that you have on your phone is the most useful thing. You consult it wherever you want and whenever you want” [Guided group participant]	
**The experience with the guide**			
	Support	“[...]the interaction itself was effective in the sense that it explained things to me when I had doubts, that it directed me, maybe, when I didn’t quite understand the task, it directed me well[...]” “[...]giving me suggestions, helping me even on how not to give up-because maybe there were also harder moments-thus also giving me alternatives and suggestions to see the path in a different way, make it lighter[...] I had a great time”	
	Personalization of the conversation	“[...]the redundancy of the messages[...] sometimes it almost felt like a copy and paste of the messages and not an actual interaction[...] I found it a little depersonalized[...] I would have found it more enjoyable if it was personalized[...]” “I saw that the person who was on the other side—I don’t like to say ‘Joy the guide!’—I still found her to be a person who each time, with respect to my mood, to how my week had gone, has put herself into my shoes, into my being, into my experience[...]”	

#### Participant’s Personal Experience With the Overall Intervention

Coherent with the *self-observation and activities evaluation* subtheme reported in Table 4, participants reported a positive personal experience as they seemed aligned with the content of the intervention, thus learning to appreciate the value of self-observation (regarding behavior and emotions) and how this can influence how they feel. For instance, the participant from the unguided group reported as follows:

[...]it [the intervention] was helpful, because it may not seem like it but by writing down daily what you do... you notice things that maybe normally you wouldn’t notice, or [discover] free time that you can spare, which maybe you didn’t even think about...

Coherently, a participant from the guided group affirmed as follows:

[...]in relation to well-being, it definitely helped me understand[...] where to aim to get better... being at home during pregnancy, I rediscovered some hobbies that helped me... it helped me to not be so focused on negative things[...] to identify something positive to do day-by-day, to be able to accomplish little goals that [might not be important for others] but for me at that moment were important.”

Participants seem to have internalized the “message” that the intervention wanted to transmit. When asked whether they believed what they had done during the intervention could be useful to them in the future, participants reported already having integrated what they have learned into their daily life, for instance, by starting “[...]to keep a journal[...]” (a participant from the unguided group), even after pregnancy. Notably, a participant from the guided group reported as follows:

[E]ven in conditions of not pregnancy... it might be a good method at other times in life when one may experience difficulties.

Referring to the subtheme of *learning the “method” and its application in the future,* this highlights that the intervention content was able to provide the participants with a broader method useful to support their psychological adjustment and well-being both during pregnancy and in the future:

[...]it’s like, mentally, I got into this mode of planning something nice to do, to be able to have that weekly commitment that I like, to ask somebody to do it with me. [These] may be small things, but I’m sure it will help me in the future as well.

Guided group participant

In this regard, and referring to the *effort subtheme,* participants reported that 6 weeks of intervention were enough to this end, while the subsequent optional 3 weeks were perceived as a bit redundant and excessive. In addition, they stressed the effort required by the intervention overall, affirming, for instance, as follows:

[A]s the weeks went on[...] I found it more burdensome[...] filling out the daily form chart [daily monitoring form] every day with the activities perhaps, as time went on, was challenging, in the sense that you have to stop and really take [your] time and be consistent, when as the pregnancy progresses maybe other thoughts take over and you can’t quite be that consistent all the time[...]

Guided group participant

On a similar note, another participant reported as follows:

[...]the part, let’s say, the most obnoxious, difficult... is definitely the material to fill out during the last weeks. I have to tell the truth; I didn’t even finish them because I didn’t have time[...]

Guided group participant

Finally, coherent with the emerging subthemes, a further comment made by a participant from the guided group ought to be reported; she stressed that women would need to be already motivated to be able to appreciate the intervention. Indeed, she mentioned a key point, which is the need to have an adequate capacity for insight, as it might otherwise be difficult to autonomously notice the maladaptive behavioral patterns and switch to more positive ones. In this regard, the participant reported as follows:

[...]they [those following this intervention] must be people[...] who are capable of introspection... I imagine people who don’t have so much of a way of knowing themselves[...] it’s not so easy to start on such a path if you haven’t done some work on yourself first[...] also, just to have self-knowledge and say “what are my weak points?” and say “where can I go with that?”

#### UX and UE Themes

Regarding UX and UE, the *managing issues and learnability* subtheme was quite prominent. All 3 (100%) women agreed that the Moodle app was not so easy to use overall, as some participants’ approach has been that of learning how to work around what was not working to be able to continue the intervention. For instance, within an otherwise positive experience, a participant reported as follows:

...maybe the only thing that I would change, which is not really of the intervention though, [regards] more the use of the platform, is just that[...] I had trouble writing things down [for the homework...] even [the speed of the] connection when it makes you log back in to do the quiz, the page has to reload[...].

Unguided group participant

Similarly, a participant from the guided group reported as follows:

[Although she was a] *geeky chick* [as regards to the use of technology]*, it was not easy* [to use the Moodle platform]*. It took me a while to find the material[...] I had even emailed you* [the researcher conducting the interview]*... I couldn’t really understand how it worked, where they* [the materials] *were[...] I always used it from the smartphone, only a couple of times I used it from my computer[...] maybe you kind of have to learn first* [how to use the platform][...] *then I had that glitch with the quiz, and that one I found a little obnoxious, because I thought I had done the quiz[...]. it had not saved nothing. It wasn’t easy to tell when it saved what you had done, because it was a little dubious, you know, sometimes[...] it was easier to use once you had gotten the hang of it[...]*.

Altogether, this stresses the importance of the platform’s simplicity, ease of use, and related learnability regarding UX and UE. However, it should be noted, referring to the *multimedia material* subtheme, that the aesthetic of the material present, and particularly the videos and images, were much appreciated and perceived as informative.

Furthermore, coherent with the *app interventions in the future* subtheme, all 3 (100%) participants reported that they did believe that a smartphone app, being readily available, could be a valuable tool to administer this sort of intervention saying, for instance, that “...with the current use of the phone and the computer in general...an app is useful” (the participant from the unguided group) as one can “...consult it wherever and whenever*...*” (a participant from the guided group). Indeed, albeit reporting difficulties with the platform, all 3 (100%) participants had already autonomously recommended the intervention to fellow pregnant women. However, the moving force, coherent with what is reported earlier, was the intervention content:

[Although deployed through a portable tool, interventions] *help in times of transition or change. This tool* [the present digital intervention] *helps because it focuses on pleasant activities, and in such a long waiting time* [the pregnancy]*, with the struggles to organize exams, visits, what’s going to happen tomorrow[...] it helps you a little bit to[...] focus on simpler thing, that then in itself just help you every day. So yes, I would recommend it for that* [Guided group participant]

### The Experience With the Guide

Regarding the role of the guide and the guided group’s overall experience with the chat interactions, the *support* subtheme has emerged, as both participants reported the positive value of having this sort of support, whether it be practical or affective. However, intersecting with this support subtheme, the *personalization of the conversation* subtheme seemed to have weighted on participant perception of the quality of the support perceived. In particular, it seems plausible to hypothesize that as participants knew that the guide was an actual person, the way of talking of the guide in the free sections of their protocol guided the participants’ perception of their capacity for empathy and perception of getting in tune with them. Indeed, while 1 (33%) of the 3 participants reported the intervention to be not personalized enough, 1 (33%) reported almost the opposite. More specifically, a participant reported as follows:

[...]the interaction itself was effective in the sense that it explained things to me when I had doubts, that it directed me, maybe, when I didn’t quite understand the task, it directed me well. Um, the part that I definitely didn’t like was the redundancy of the messages because sometimes it almost felt like a copy and paste of the messages and not an actual interaction[...] I found it a little depersonalized[...] I would have found it more enjoyable if it was more personalized.

The other, instead, reported expecting from the beginning a set of predetermined questions from the guide, particularly as some questions were indeed repeated each week; however, the participant reported about the guide as follows:

[S]aw that the person who was on the other side, I don’t like to say “Joy the guide”- I found them to be a person who each time, with respect to my mood, to how my week had gone, has put herself into my shoes, into my being, into my experience, giving me suggestions, helping me even on how not to give up-because maybe there were also harder moments-thus also giving me alternatives and suggestions to see the path in a different way, to make it lighter. So, I had a great time!

Such difference in the perception of the guide and of their helpfulness seems even more plausible when considering that a participant (who did not agree to the semistructured interview) had finished viewing the modules on Moodle until T6 but did not continue with interactions with the guide after T3.

## Discussion

### Principal Findings

This study aimed to investigate the feasibility of the BATD-R protocol [31] that was structured as an internet-based self-help intervention and deployed to pregnant women with subclinical depression symptoms, while further comparing its guided versus unguided versions. Such evaluations had the associated purpose of collecting the feedback needed to refine the intervention, thereby allowing its co-design and adaptation; moreover, it represents the first instance where the replication of the in-person BATD-R into a digital format has been empirically measured and evaluated, yielding valuable insights and shortcomings useful for future research.

Overall, results showed that this first version of the digital BA intervention, JuNEX, as a “replica” of the original BATD-R protocol [31], is not feasible to be implemented in a digital e-learning format. Specifically, data have highlighted the need to lighten the intervention and reduce the effort required for homework. Despite the perceived excessive effort, participants appreciated the content and purpose of the intervention, which allowed them to “take a moment” for themselves and understand “where to aim to get better.” Indeed, albeit the intervention protocol originated from a behavioral framework, its focus on the person’s everyday activities, the evaluation of one’s own experiences, and how these are linked to how behaviors and emotions mutually influence each other configure in line with third-generation cognitive behavioral therapies [54]. These therapies prioritize holistic enhancement of psychological and behavioral processes related to health and well-being [55], emphasizing adaptive coping methods and increasing experiential and contextual awareness [55,56]. Such an approach is particularly relevant for nonclinical populations (which are more diverse than clinical populations), as they allow for a more transversal relevance and application of the coping methods promoted, thus making the intervention especially valuable in preventive terms. Given that this study focused on women with subclinical depression symptoms, interventions emphasizing the awareness of psychosocial functioning and the interplay between emotions and behaviors may be more beneficial than targeting specific, limited areas of functioning and distress. This broader approach could enhance women’s capacity for adjustment and could be applicable to difficult situations beyond pregnancy-related challenges.

Such explanations serve to emphasize that consistent with women’s feedback, the intents of the intervention per se, and thus its content as well as homework purposes, ought to be maintained as they were found pertinent to the pregnancy situation and were appreciated by women; however, data also stress the need to structure the intervention so that it can be deployed with greater ease and the need to shorten it to maximum of 6 weeks. In this regard, the data do point to satisfactory usability and UX as pivotal aspects of the intervention feasibility, which was not provided either by the Moodle platforms or the Google Docs used. These platforms were used in line with the preliminary and exploratory nature of this study, allowing the first structuring of the BATD-R protocol in a digital setting in a time- and cost-efficient manner; this has favored the co-design of the future development of the intervention by developing a more advanced and refined application after having collected some initial pregnant women’s feedback.

When evaluating the usability of a digital solution, 5 main aspects are to be considered as follows [57]: (1) the simplicity of use experienced by users when learning how to use a digital tool, (2) the number of mistakes they make to do a certain action correctly, (3) the effectiveness with which users interact with a digital tool, (4) the perceived satisfaction with the UX, and (5) the memorability of how to use a digital tool after having been exposed to it. In addition to this, and particularly linked to both the effectiveness and perceived satisfaction just mentioned, is the more specific UE, thus linked to the subjective experience of the user and subsuming affective, cognitive, and behavioral components [25]. Considering the UX and UE evaluated in this study, data suggest that usability and UE were mediocre, yet not scarce. Thus, there was some appreciation for the tools used by the participants who had completed the interventions; however, the simultaneous high dropout and overall low recruitment of participants strengthen the idea that the more positive feedback given is somewhat linked to the a priori internal motivation of the participants in following the intervention. Participants themselves have indeed underlined that to follow the intervention as it is, to be able to bear the effort required by it, and to work around the limits of the platform used, women would need to be highly motivated on their own.

Coherent with the importance of the users’ motivation to properly follow such interventions, data do suggest that the guided group showed greater adherence and were overall more willing to finish the intervention compared to the unguided group. A part of the guides’ job was indeed to motivate women to favor compliance and adherence, and as women knew that they were interacting with a psychologist, this can be thought to have further increased their motivation. However, the practical challenges of having to set a date and time for the interactions are the limiting factors and so are the individual differences related to the guides’ different writing modalities. In this regard, a step further in this direction might be the design and inclusion of a conversational agent to provide guidance and support during the digital intervention. Conversational agents can greatly favor the personalization of the user-system interactions while fostering scalability by requiring a much-reduced workforce for the intervention administration [58]. Existing literature has highlighted that conversational agents might be valuable tools to foster intervention adherence by favoring engagement and involvement, thereby supporting the overall UX [58,59] and allowing for a more immersive experience. Using a conversational agent is expected to further reduce time constraints for both patients and clinical professionals while also reducing health care costs in the long run. Within the perinatal context, future studies should thus evaluate the potentiality of including a conversational agent within digital interventions to ultimately support women’s motivation and engagement as well as their compliance and adherence to the intervention, allowing them to use such digital solutions more freely while still giving the feeling of a more personalized experience.

Coherently, and in line with past evidence [60-62], women have expressed a desire for web-based solutions that support their psychological well-being. Furthermore, the data in this study seem to suggest that women might prefer app-based solutions, as almost all (3/4, 75%) participants that completed at least up to T6 used the app version instead of the web version to follow the intervention. Compared with web-based programs, interventions deployed through a smartphone app are much more readily available wherever and whenever, thus being overall easier to use and access.

### Limitations

This paper reports the first-phase study’s findings, highlighting valuable insights and several limitations that can guide future research and the development of digital interventions. First and foremost is the inherent challenge of having endeavored to replicate or simulate an in-person intervention digitally, a task that resulted in an ineffective outcome in our case. It is crucial to acknowledge that digital solutions offer a unique environment and set of possibilities that distinguish them from face-to-face interactions. Attempting to mirror traditional methods within this digital landscape may fall short of fully capitalizing on the advantages that digital interventions can bring. With our findings, we thus strengthened the idea that digital interventions should not be mere replicas of their in-person counterparts; rather, they should harness distinctive strengths and capabilities. This challenge underscores the need for innovation and adaptation, as well as a recognition that a direct translation of traditional methods may not always yield optimal results in the digital sphere. Future studies should first try to conduct workshops with end users in which the in-person protocol is administered to discuss feasible changes to the intervention, thus singling out the intervention’s main principle and appreciated practices and then adapting them to the digital format. Furthermore, a subsequent limitation of the study overall is the limited sample size, which has prevented the possibility of computing any statistical comparisons between the 2 groups as well as generalizing findings. Nonetheless, given the preliminary and exploratory nature of this study, aimed at setting the base for the co-design of the final intervention, the data collected were still able to provide valuable insights directing the refinement and future developments of the intervention. Further limitations are attributed to the platforms used, as they are created with different purposes than what they were used for in this study. This has warranted structuring the intervention content based on the functionalities of this platform instead of creating a tool tailored to the intervention requirements; therefore, future studies are advised to avoid using such tools to administer and evaluate digital intervention, even in the first-phase studies such as this one. However, it should still be emphasized that such platforms (ie, Moodle and Google Documents) have allowed to “prototype” the intervention in a time- and cost-efficient manner while collecting the information needed to refine the intervention and to create a specific app that can meet the requirements and preferences of the users.

### Conclusions

This study had the purpose of evaluating the feasibility of the BATD-R protocol [31] structured as an internet-based self-help intervention among pregnant women with subclinical depression symptoms while comparing its guided versus unguided versions. A subsequent goal was the collection of women’s feedback and perceptions of the intervention content through a prototyped version based on an e-learning platform to allow the co-design of the final intervention.

Overall, the ease with which the intervention can be followed has emerged as a central component to account for in the future developments of the intervention, in terms of the intervention itself as well as the platform used to administer it. The greater effort perceived was related to the homework, whereby women did emphasize that performing them was effortful and time-consuming. Even so, comparing the 2 versions of intervention, the guided version was more well-received than the unguided version. Nonetheless, both groups expressed satisfaction with the intervention’s content and felt that it was relevant to their personal experiences with pregnancy.

## Abbreviations

BA: behavioral activation
BATD-R: Behavioral Activation Treatment for Depression-Revised
DSM-IV: Diagnostic and Statistical Manual of Mental Disorders (Fourth Edition)
MARS: Mobile Application Rating Scale
PHQ-9: Patient Health Questionnaire-9
UE: user engagement
UX: user experience
